# An Adult with a Remnant Urachus Anomaly Diagnosed in the Emergency Department

**DOI:** 10.1155/2018/6051871

**Published:** 2018-08-14

**Authors:** Alan Lucerna, James Lee, James Espinosa, Risha Hertz, Victor Scali

**Affiliations:** ^1^Department of Emergency Medicine, Rowan University SOM/Jefferson-Stratford, NJ, USA; ^2^Penn Medicine, Gibbsboro, NJ, USA; ^3^Department of Emergency Medicine, Rowan University SOM, USA

## Abstract

The urachus is a midline tubular structure that stretches from the apex of the bladder and connects to the umbilicus. Urachal remnants result from incomplete regression of the fetal urachus in infancy. We report the case of a 21-year-old male who presented to the emergency department with purulent drainage from his umbilicus in association with a chronic intermittent “pulling sensation” in the umbilicus and suprapubic areas. An infected urachal remnant was diagnosed and was treated with an oral antibiotic and ultimately with outpatient excision of the remnant. Such cases are rare but have the potential to progress to sepsis. In addition, chronic inflammation can lead to neoplastic transformation (adenocarcinoma). Urachal remnant infections can be considered in adults with umbilical purulent drainage. We propose that the “pulling sensation” described may be a clue to the diagnosis in some patients in which the urachal remnant is attached to the bladder and that the sensation was due to the mechanical connection between the bladder and the umbilicus. The sensation resolved postremoval status of the remnant. This does not appear to have been previously proposed in the literature.

## 1. Introduction

The urachus is a midline tubular structure that stretches from the apex of the bladder and connects to the umbilicus. Also called the median umbilical ligament, the urachus is a vestigial remnant of two embryonic structures: the cloaca and the allantois [1]. The fetal urachus is obliterated as part of the normal development of infancy. Urachal remnants result from incomplete regression of the fetal urachus [2]. Such remnants can become infected and can present with umbilical discharge and therefore could be diagnosed as an umbilical cellulitis. However, an infected umbilical remnant has the potential for progression to cellulitis. There is also the potential for neoplastic transformation.

## 2. Case Report

A 21-year-old male presented to an emergency department (ED) with a two-day history of purulent drainage from his umbilicus. He denied pain. He reported that he had experienced a similar episode one year prior which was diagnosed in an ED as umbilical cellulitis and resolved with oral antibiotics. Interestingly, the patient noted that since childhood he had recurrent episodes of what he described as a “pulling sensation” in the umbilicus and suprapubic areas. The patient had seen multiple specialists regarding the sensation but the workups had been unremarkable.

The patient had no pertinent medical or surgical history. The family and social history were unremarkable. He did not take any medications and denied drug and alcohol use.

Vital signs on presentation were blood pressure 139/82 torr, heart rate 55 beats per minute, respiratory rate 15 breaths per minute, and temperature 98.5 degrees F. (oral temperature) with a pulse oximetry reading of 100% on room air.

Yellowish discharge was noted from center of the umbilicus. The periphery of the umbilicus was erythematous with mild tenderness to palpation. The physical examination was otherwise unremarkable.

The complete blood count and basic metabolic panel were within normal limits. The urinalysis was unremarkable.

**Figure 1:**
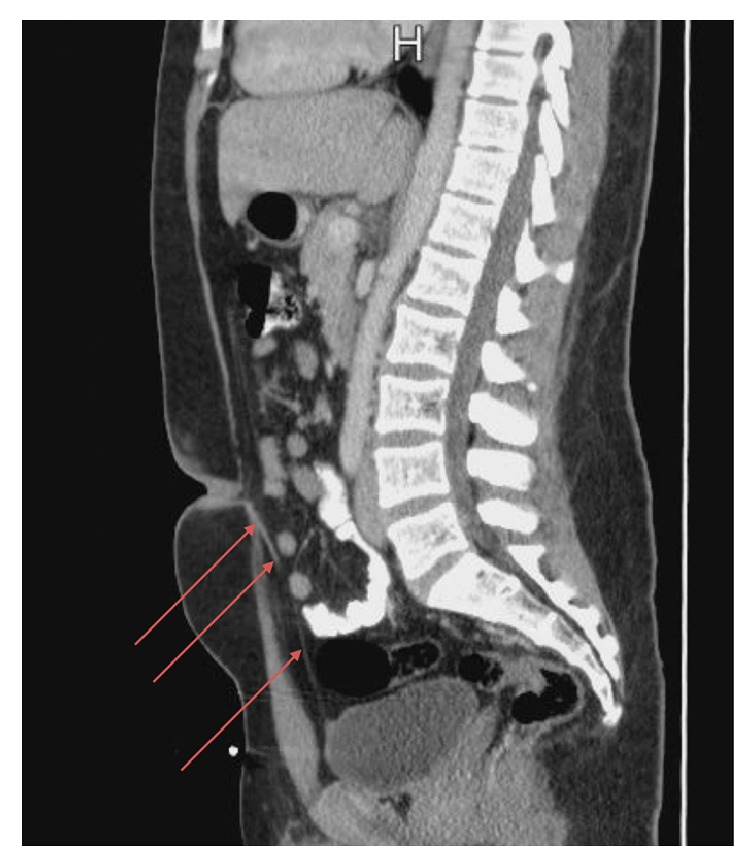
Computed tomography (CT) scan of the abdomen and pelvis sagittal plane showing a urachal remnant (red arrows) arising from the anterior/superior margin of the bladder and extending to the umbilical region. The remnant consists of a thin fibrous band of tissue measuring up to 4.6 mm in thickness near the umbilicus although measures as narrow as 2.0 millimeter along its course.

**Figure 2:**
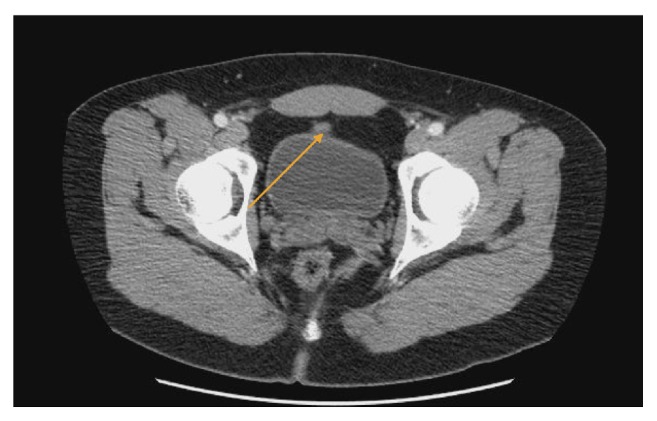
Computed tomography (CT) scan of the abdomen and pelvis axial section showing urachal remnant anterosuperior midline of the bladder (orange arrow).

A computed tomography (CT) scan of the abdomen and pelvis with oral and intravenous contrast revealed an urachal remnant arising from the anterior/superior margin of the bladder and extending to the umbilical region. The remnant consisted of a thin fibrous band of tissue measuring up to 4.6 mm in thickness near the umbilicus. The band narrowed to a minimum of 2 mm along its course. No umbilical fluid collection was identified. The abdominal fat posterior to the umbilicus showed no inflammatory reaction (Figures 1 and 2).

An infected umbilical-urachal sinus was suspected. The patient was treated with oral antibiotics since the patient had success with a similar presentation a year prior to the ED visit. He was instructed to follow up with a urologist in the outpatient setting for definitive treatment. His infection was resolved. He later underwent an excision of the urachal remnant. Histology showed no evidence of malignant transformation.

## 3. Discussion

Urachal remnant abnormalities are uncommon in adults and are twice as likely in men than in women [1–4]. The incidence of congenital urachal anomalies diagnosed at or shortly after birth is reported to be less than 2 cases per 300,000 pediatric admissions [3]. A considerably higher number of cases (1 case per 5000) were identified incidentally in pediatric autopsies, suggesting that many patients with this anomaly are clinically asymptomatic [3]. Symptomatic urachal anomalies most commonly present between the ages of 20 and 40 years [3]. Our patient complained of a long-standing “pulling sensation” in the lower abdomen and previously had numerous workups by different specialists, all of which were nondiagnostic.

In addition to umbilical area pain, patients may report nonspecific symptoms such as suprapubic abdominal discomfort, hematuria, dysuria, urinary frequency, or urgency [1].

The most common complication of a urachal anomaly is infection [4]. There are various types of urachal abnormalities. The entire tract can be patent, or various types of cysts and sinus tracts can remain. The urachal cyst is the most common type of adult urachal abnormality [2]. Infected cysts can present with a tender midline mass below the umbilicus. Many types can present with umbilical discharge [5]. Hirose et al. described a case of a middle-aged man who presented with umbilical discharge where an abscess had formed at the urachal remnant, requiring drainage and intravenous antibiotics [6]. Our patient presented with two days of purulent drainage from the umbilicus and a long-standing history of a “pulling sensation” from the suprapubic region. The sensation was exacerbated by urination. This was his second such presentation. The cause of this sensation is not clear.

We suspect that the sensation was due to the mechanical connection between the bladder and the umbilicus. The sensation resolved postremoval status of the remnant.

In reference to imaging, although ultrasound has been shown to be helpful, the definitive diagnostic imaging modality is computed tomography [7].

Early detection of these anomalies can help optimize treatment plans to prevent subsequent complications such as infection. Sepsis can be a complication [3].

The innermost lining of a urachal remnant is comprised of transitional epithelium. Chronic inflammation can lead to metaplasia, principally adenocarcinoma [5, 7].

Though rare, emergency physicians may benefit from having such anomalies in their differential when evaluating patients, especially patients older than infants, who present with an umbilical discharge. If diagnosed in the ED, urological evaluation is warranted for further evaluation and definitive treatment. We propose that the “pulling sensation” described may be a clue to the diagnosis in some patients in which the urachal remnant is attached to the bladder. We suspect that the sensation was due to the mechanical connection between the bladder and the umbilicus. The sensation resolved postremoval status of the remnant.

This does not appear to have been previously proposed in the literature.

## 4. Conclusion

Urachal remnant infections can be considered in adults with umbilical purulent drainage. Early diagnosis and treatment may prevent subsequent complications such as infection, including sepsis as well as the potential long-term complication of adenocarcinoma.
